# My Second Thoughts About Abdominoplasty: A Video Interview With Dr Francisco Villegas

**DOI:** 10.1093/asjof/ojad106

**Published:** 2023-11-29

**Authors:** Francisco Villegas, Lorne King Rosenfield

Welcome to *ASJ*'s Second Thoughts on First Thoughts. We are treated today to a truly profound second thought by a genuine surgical innovator, Francisco Villegas, from Cali, Colombia. Francisco will enlighten us as to how, when, and why he turned the traditional abdominoplasty on its head with his counter-intuitive vertically oriented abdominal fascial plication. And it is worth noting that Dr Villegas’ “aha” moment came while working on, not another cosmetic abdominoplasty, but rather a reconstructive hernia case! (Video). Once more proving the value of all aesthetic surgeons striving to fashion a quiver replete with strong reconstructive skills. Enjoy.

## Supplementary Material

ojad106_Supplementary_Data

